# Clear cell sarcoma of the soft parts arising in the rectus abdominis in a child – aspiration cytology of a rare case

**DOI:** 10.1186/1742-6413-4-15

**Published:** 2007-07-15

**Authors:** Paari Murugan, Debdatta Basu, Surendra Kumar, Sadasivan Jagadish

**Affiliations:** 1 Dept. of Pathology, Jawaharlal Institute of Postgraduate Medical Education and Research, Pondicherry, India; 2 Dept. of Surgery, Jawaharlal Institute of Postgraduate Medical Education and Research, Pondicherry, India

## Abstract

**Background:**

Clear cell sarcoma of soft parts is most commonly found associated with the tendons and aponeuroses of distal extremities in young adults with a peak incidence in the third decade. Location in the abdominal wall and in a child is very rare.

**Case presentation:**

A nine-year-old female child presented with a swelling in the anterior abdominal wall in the suprapubic region. Fine needle aspiration revealed predominantly discrete cells with loose clustering at places. The cytoplasm was abundant, finely granular, and eosinophilic with some cells exhibiting clear vacuolated zones. No pigment was seen. The nuclei were rounded and eccentrically placed with a striking single eosinophilic macro nucleolus present in all the cells. Taking into consideration, the history, age of the patient, location of the tumor and absence of melanin pigment, a diagnosis of soft tissue sarcoma was made, the differential including Clear cell sarcoma. This was subsequently confirmed on histopathological examination and immunohistochemistry

**Conclusion:**

The atypical presentation of the case made the cytological diagnosis rather challenging. Clear cell sarcoma should be considered when cytology of a soft-tissue tumor shows uncharacteristically high cellularity and relatively uniform cells with macronucleoli.

## Background

First described by Enzinger in 1965, clear cell sarcoma of the soft parts (CCS) is also called Melanoma of soft parts [1,2]. Over the years, CCS has been established as a distinct clinicopathological entity with the tumor specific translocation t (12; 22) (q13; q12) or the chimeral EWS/ATF1 gene, identified in 50–75% of the cases [3]. Though it shares the immunohistochemical and ultra structural features of malignant melanoma, CCS differs by virtue of its location, age of presentation and the cytogenetic abnormality [4].

CCS is most commonly found associated with the tendons and aponeuroses of distal extremities in young adults with a peak incidence in the third decade [2]. CCS is uncommon below the age of ten years [5].

While several large series have described in detail the histopathological features of CCS [1,2,6], cytomorphological features of CCS have been recorded in a few small reports and two case series [7-11]. The diagnosis of CCS on fine needle aspiration cytology requires a high degree of suspicion given its rarity of presentation and overlap of morphology with malignant melanoma, metastatic adenocarcinoma and other soft tissue sarcomas like alveolar soft part sarcoma, extra renal rhabdoid tumor, epithelioid sarcoma and synovial sarcoma [7,8].

We describe the cytological features of CCS in a child presenting in the rectus abdominis tendon, an extremely rare location.

## Case presentation

### Brief clinical summary

A 9-year-old female child presented with complaints of pain in the right iliac region just superior to the pubic symphysis associated with painful micturition for a period of six months. On examination, there was a tender firm irregular 5 × 3 cm mass felt near the insertion of the rectus abdominis tendon. The skin appeared normal and no lymph nodes were palpable. CT scan showed an irregular non enhancing 5 × 5 × 2.5 cm hypodense lesion in distal right rectus abdominis. No evidence of calcification or intraabdominal extension was seen. The bladder appeared normal.

### Cytological findings

A freehand aspiration guided by palpation was done using a 22 gauge needle and 10 ml syringe. Both air dried and 95% ethanol fixed smears were prepared and stained by the May Grunwald Giemsa and Papanicolaou respectively.

**Figure 1:**
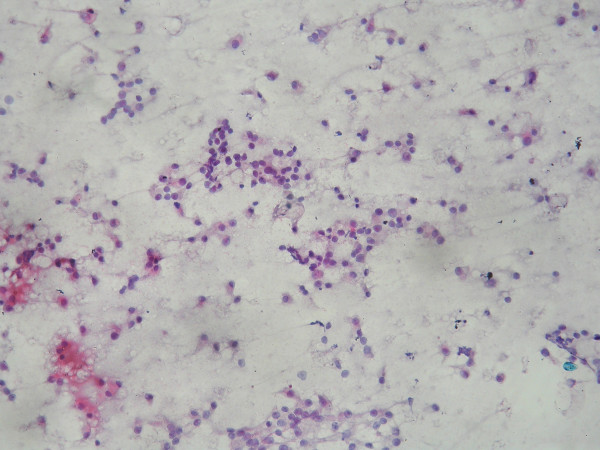
Moderate to highly cellular smear with predominantly discrete cells. Loose clusters are also seen. The background is relatively clean. Papanicolaou stain × 200.

**Figure 2:**
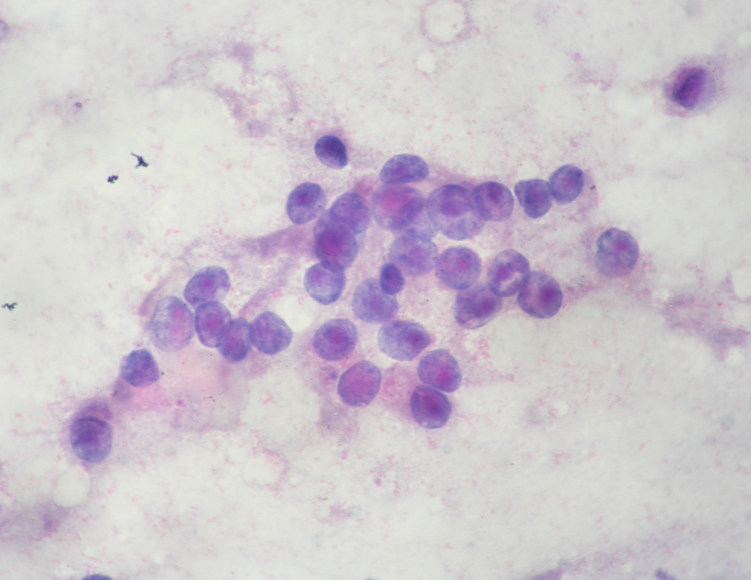
Loose cluster of epithelioid cells with abundant granular cytoplasm, rounded nuclei with mild to moderate anisokaryosis and prominent eosinophilic macro nucleolus. Note the micro acinus like formation in the centre. Papanicolaou stain × 1000.

The smears were moderately cellular with a relatively clean background, lacking inflammation or necrosis [Fig 1]. The cells were predominantly discrete with loose clustering at places and occasionally showed evidence of micro-acini like formations [Fig 2].

**Figure 3:**
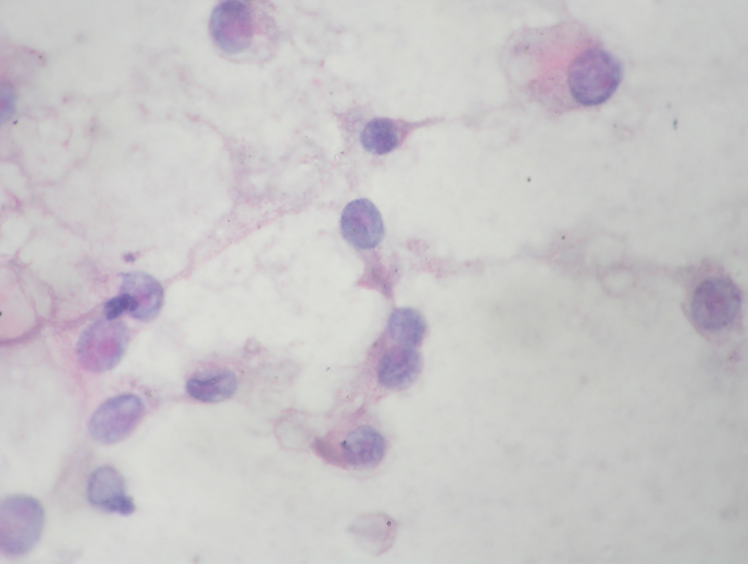
Discrete cells showing abundant granular cytoplasm with vacuolation. Papanicolaou stain × 1000.

The cytoplasm was abundant, finely granular, and eosinophilic with some cells exhibiting clear vacuolated zones. No pigment was seen. The nuclei were rounded and eccentrically placed with a moderate degree of anisonucleosis. The chromatin was fine, evenly dispersed. A striking single eosinophilic macro nucleolus was noted in all the cells [Fig 3]. None of the nuclei showed inclusions. Mitotic figures were absent.

Based on these features and taking into consideration, the history, age of the patient, location of the tumor and absence of melanin pigment, a diagnosis of soft tissue sarcoma was made, the differential including CCS. Following FNA interpretation, a wide local excision was done.

### Gross and microscopic histologic findings

The resected specimen was nodular with attached skeletal muscle and measured 5 × 5 × 2.5 cms. The cut surface showed a firm fleshy homogenous gray white mass appearing to be in continuum with the surrounding skeletal muscle/tendon [Fig 3}.

**Figure 4:**
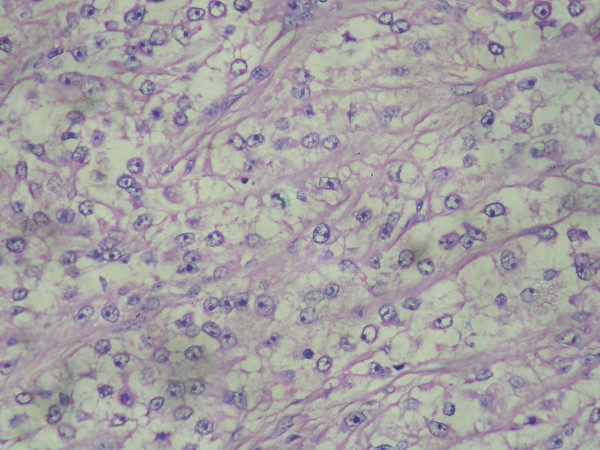
Thin fibrous septa dividing cells arranged in a nesting/alveolar pattern with prominent nucleolus and clear cytoplasm. Hematoxylin and Eosin × 400.

Hematoxylin and eosin stained sections from the tumor revealed nests and alveoli like arrangement of the cells separated by thin fibrous septa intimately associated with the peripheral muscle and dense tendinous connective tissue. Predominantly the cells were polygonal with clear cytoplasm containing PAS positive material. The nuclei were rounded; relatively uniform, with mild, if any, pleomorphism, vesicular chromatin and prominent nucleolus. (Fig 4) Foci of cells with granular eosinophilic cytoplasm were also seen. No evidence of mitoses, hemorrhage, necrosis or pigment was made out.

A stain for reticulin further illustrated the nesting pattern. Fontana Masson stain for melanin pigment was negative.

**Figure 5:**
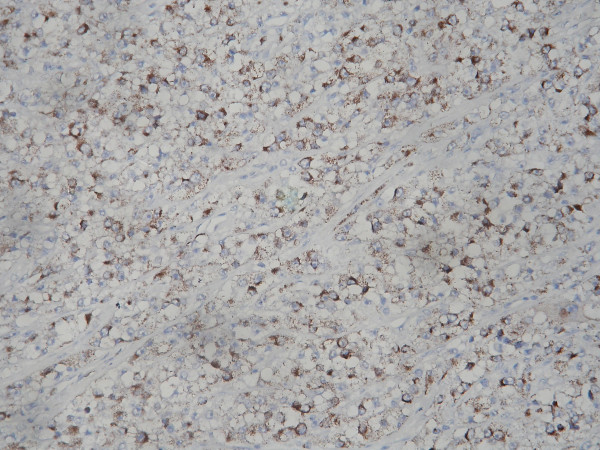
Immunohistochemistry with HMB 45 showed the tumor cells exhibiting cytoplasmic positivity. IHC – Streptavidin Biotin × 200.

**Figure 6:**
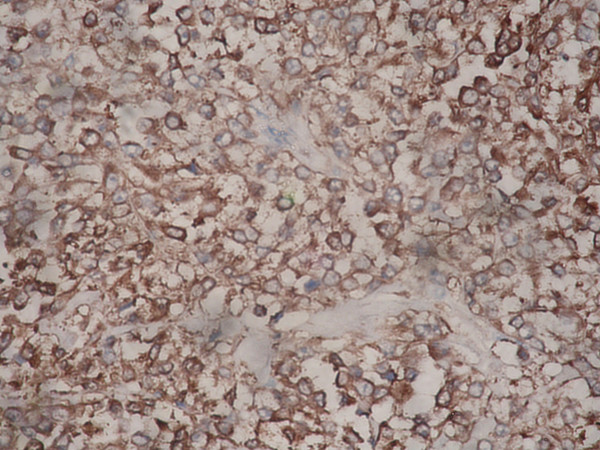
The tumor cells expressed a strong positivity for Neuron specific Enolase (NSE). IHC – Streptavidin Biotin × 400.

**Figure 7:**
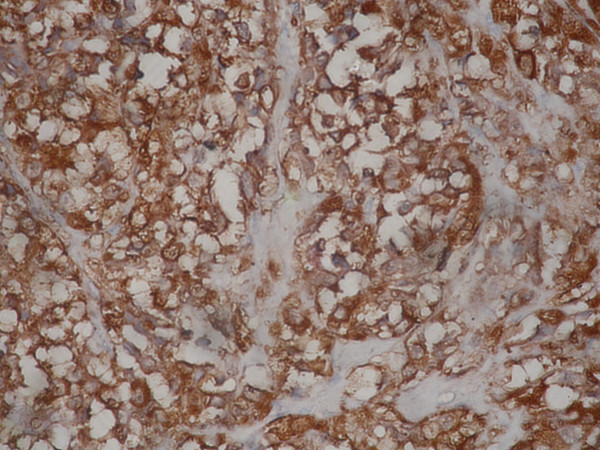
The tumor cells also were positive for S100. IHC – Streptavidin Biotin × 400.

The morphology was suggestive of CCS and the diagnosis was confirmed by immunohistochemistry. The tumor cells stained positive for HMB 45 [Fig 5], Neuron specific enolase (NSE) [Fig 6] and S100 [Fig 7]. Desmin, CD99 and Melan A were negative. Cytogenetic study could not be done in the present case.

## Discussion

CCS is a rare tumor representing around 1% of all soft tissue sarcomas [2]. It is also known as melanoma of soft parts, acknowledging the similarity in the neural crest origin as well as in the cytological, immunohistochemical and ultrastructural features of both tumors [2,4]. Nevertheless, the unique t (12; 22) (q13; q12) translocation, younger age of presentation, location in deeper soft tissues associated with tendons/aponeuroses, absence of epidermal involvement and predilection to the extremities help in distinguishing it from malignant melanoma [4].

CCS mainly affects young adults between the ages of 20 and 40 years [2,6]. Its occurrence in the pediatric age group is rare, with only 2% of the reported cases having been reported below the age of ten years [2,5].

The usual principal sites of the neoplasm are in the extremities, especially the region of the foot and ankle, followed by the knee, thigh and hand [2,7]. Nevertheless, a few reports of CCS have spoken of unusual sites which include lung [7], chest wall [12] cervical spinal cord [13], scapula [10,14], retroperitoneum [15]. Interestingly, of the 28 pediatric patients reported by Ferrari, eight occurred in sites other than extremities including one case in the abdomen [5]. The exact location in the abdomen was however not described by the authors [5]. To the best of our knowledge, involvement of the rectus sheath has only been reported once before [16].

There are a few cytological reports of CCS, including two case series' comprising of eleven and nine cases by Creager et al and Caraway et al [7,8]. The features which serve as common denominators in these reports are – the abundant cellularity (in contrast to other soft tissue sarcomas with epithelioid morphology simulating CCS), presence of both clusters and dispersed discrete polygonal, rarely fusiform, cells with abundant clear to finely granular cytoplasm, eccentrically placed round hyperchromatic nuclei showing moderate degree of anisonucleosis, single prominent nucleolus and occasionally multiple smaller nucleoli [7-11]. Intranuclear cytoplasmic inclusions and cytoplasmic pigment have also been described though not present in our case [17]. A rare granular cell variant has also been recognized [7]. Nguyen et al have reported of a case of CCS with marked cellular cohesiveness and moulding, which has not been identified in any of the reported cases including ours [18]. In fact dispersion and decreased cohesiveness have been the 'defining cytological features' of these neoplasms [7-11].

Given the cytomorphological appearance and age of presentation of the tumor, a differential diagnosis of epithelioid sarcoma, alveolar soft part sarcoma and extra renal rhabdoid tumor may be entertained on cytological smears [19]. Metastatic adenocarcinoma can be considered in older patients, especially if acinar formations are seen [7,10]. Immunocytochemisty, electron microscopy and lack of the signature translocation will help in differentiating these tumors from CCS and may be resorted to in problematic cases.

Immunohistochemically, 80% of tumors contain cells that express S-100 protein, and more than three-fourths are HMB45-positive [9]. Melan A, NSE and Leu 7 have also been found positive. Ultra structurally, melanosomes in varying stages of development are seen in a majority of cases [4]. In the present case, S100 and HMB45 were strongly positive. The tumor cells also stained positive for NSE while the other melanoma associated marker, Melan A was negative. Ultra structural studies were not done.

The prognosis for these tumors is poor with high incidence of recurrence and metastasis. Necrosis and a larger tumor size (>5 cms) is associated with a high rate of distant failure [6]. Prognosis is also significantly better for patients whose tumors arise in the extremities rather than at other sites where complete tumor excision is more difficult. However, age does not represent a statistically significant prognostic factor [5]. The treatment is primarily surgical. Our patient has been on regular follow up for the past five months and has not presented with any complications till date.

## Conclusion

Cases of CCS are uncommon below the age of ten years. In addition, the location of the tumor in the present case – the rectus abdominus tendon, is extremely rare. Cytologically, the tumor may simulate other soft tissue sarcomas with epithelioid morphology. The diagnostic difficulty is thus compounded in cases with unusual clinical presentation, though the cytological features of high cellularity with dyscohesive cells showing clearing of cytoplasm and macronucleoli often offers a substantial clue in arriving at an accurate diagnosis.

## References

[B1] Enzinger FM (1965). Clear cell sarcoma of tendons and aponeuroses:an analysis of 21 cases. Cancer.

[B2] Chung EB, Enzinger FM (1983). Malignant melanoma of soft parts. A reassessment of clear cell sarcoma. Am J Surg Pathol.

[B3] Hiraga H, Nojima T, Abe S, Yamashiro K, Yamawaki S, Kaneda K, Nagashima K (1997). Establishment of a new continuous clear cell sarcoma cell line: morphological and cytogenetic characterization and detection of chimaeric EWS/ATF-1 transcripts. Virchows Arch.

[B4] Langezaal SM, Graadt van Roggen JF, Cleton-Jansen AM, Baelde JJ, Hogendoorn PC (2001). Malignant melanoma is genetically distinct from clear cell sarcoma of tendons and aponeurosis (malignant melanoma of soft parts). Br J Cancer.

[B5] Ferrari A, Casanova M, Bisogno G, Mattke A, Meazza C, Gandola L, Sotti G, Cecchetto G, Harms D, Koscielniak E, Treuner J, Carli M (2002). Clear cell sarcoma of tendons and aponeuroses in pediatric patients: a report from the Italian and German Soft Tissue Sarcoma Cooperative Group. Cancer.

[B6] Lucas DR, Nascimento AG, Sim FH (1992). Clear cell sarcoma of soft tissues. Mayo Clinic experience with 35 cases. Am J Surg Pathol.

[B7] Creager AJ, Pitman MB, Geisinger KR (2002). Cytologic features of clear cell sarcoma (malignant melanoma) of soft parts. A study of fine-needle aspirates and exfoliative specimens. Am J Clin Pathol.

[B8] Caraway NP, Fanning CV, Wojcik CM, Staerkel GA, Benjamin RS, Ordonez NG (1993). Cytology of malignant melanoma of soft parts: fine needle aspirates and exfoliative specimens. Diagn Cytopathol.

[B9] Tong TR, Chow TC, Chan OW, Lee KC, Yeung SH, Lam A, Yu CK (2002). Clear-cell sarcoma diagnosis by fine-needle aspiration: cytologic, histologic, and ultrastructural features; potential pitfalls; and literature review. Diagn Cytopathol.

[B10] Kumar N, Das PM, Jain S, Sodhani P, Gupta S (2003). Melanoma of the Soft Parts: Diagnosis of Metastatic and Recurrent Tumors by Aspiration Cytology. Diagn Cytopathol.

[B11] Almeida MM, Nunes AM, Frable WJ (1994). Malignant melanoma of soft tissue. A report of three cases with diagnosis by fine needle aspiration cytology. Acta Cytol.

[B12] Suehara Y, Yazawa Y, Hitachi K, Terakado A (2004). Clear cell sarcoma arising from the chest wall: a case report. J Orthop Sci.

[B13] Jiggins M, Prosser G, Jackowski A (2002). Clear cell sarcoma (malignant melanoma of soft parts) presenting as cervical myelopathy. Br J Neurosurg.

[B14] Kazakos CJ, Galanis VG, Giatromanolaki A, Vrettas D-AJ, Sivridis E (2006). Clear cell sarcoma of the scapula. A case report and review of the literature. World J Surg Oncol.

[B15] Katabuchi H, Honda R, Tajima T (2002). Clear cell sarcoma arising in the retroperitoneum. Int J Gynecol Cancer.

[B16] Finley JW, Hanypsiak B, McGrath B, Kraybill W, Gibbs JF (2001). Clear cell sarcoma: the Roswell Park experience. J Surg Oncol.

[B17] Shabb NS, Boulos F, Tavvil A, Hussein M, Hourani M (2003). Clear Cell Sarcoma (Malignant Melanoma of Soft Parts): Fine-Needle Aspiration Cytology of a Highly Pigmented Tumor. Diagn Cytopathol.

[B18] Nguyen GK, Shnitka TK, Jewell LD, Wroblewski JA (1986). Exfoliative cytology of clear-cell sarcoma metastases in pleural fluid. Diagn Cytopathol.

[B19] Akerman N, Domanski HA, Orell SR (2003). Tumors of uncertain or unknown origin. The cytology of soft tissue – Monographs in clinical cytology.

